# Editorial

**Published:** 1873-12

**Authors:** 


### Editorial.

### HEALTH OF THE DENTIST.

At the recent meeting of the Michigan State Dental Society, the health of the dentist was considered at some length; the great importance to all concerned of securing and maintaining the best possible state of health for the dentist, was fully conceded by all. The character of the discussion referred to gave evidence of a growing appreciation in this direction. There is further indication of progress in this matter, in the fact that there is far better health of the workers of the profession now than ten years ago. This is doubtless the result of a better apprehension of, and fuller compliance with, hygienic laws—those regulations established for our well-being—the violation or even ignoring of which works disease, suffering and death. During the last fifteen years dental practice has become more laborious and irksome than formerly, due in some measure to thefact that there is a far higher appreciation of the teeth by the public, and consequently a more urgent demand for their preservation ; and, in addition to this, dental practice now occupies a far more extended field than formerly. Operations are performed now by the profession generally that are very exhausting to the energies and strength of the dentist, that fifteen years ago were scarcely conceived of, or at least attempted. These more difficult operations bring with them a sense of additional responsibility, and in many instances a nervous tension that is very oppressive in its influence. Those who have attained much reputation for thoroughness and efficiency are usually very fully occupied, so much so that they are oftentimes broken down before they are aware of it.

Now, with the overworked dentist there is undue confinement or want of proper exercise ; inhalation of bad air ; offensive odors ; constrained and unnatural position of the body, long continued ; protracted mental and physical concentration and occupation ; a want of mental diversion ; nervous irritation ; loss of sleep ; and defective nutrition. The injurious results from each of these will be exhibited to a far greater extent by neglect. Of all men the dentist should most scrupulously regard and obey the laws of hygiene.

The dentist who is fully occupied is liable to suffer greatly from confinement or want of proper physical exercise. The demands of suffering hnmanity are constantly upon him, and these are of such a character as to call out his strictest attention ; and so, while caring for others, he forgets himself till nature cries out with sheer exhaustion.

The dentist who confines himself to his office from nine to twelve hours per day, working incessantly at his chair, without rest or change of physical exercise, should have from one to three hours appropriate exercise in the open air and sunshine every day. The character and duration of the exercise should be modified by various circumstances : such as the health and strength of the person ; intensity of his work while occupied ; the structure, location and condition of his office, etc., etc. Whilst responsibility does not rest upon the dentist as upon the physician, his physical labor is far more irksome and exhaustive ; indeed we can hardly conceive of any occupation that will draw more largely upon the physical energies than dental practice, if closely followed. We shall, perhaps, at another time consider some other points bearing upon this subject.

### CLOSE OF THE VOLUME.

This number closes the twenty-seventh volume of the DENTAL REGISTER. What it has been those who have read it know. What influence in the agregate it may have exercised for good none perhaps can tell. It, in common with others, has received criticisms, sharp, keen and many, and, on the other hand, approval, commendation and assistance, the latter coming chiefly from those who are its most careful readers and well-wishers ; the former from those who only glance at it enough to find fault, and who do nothing for its improvement. Now we would not find fault with this latter class ; if this is their mission, they should by all means fulfill it ; and we have no doubt that editorially we need all this and more for our improvement, and ability to better serve the profession, and through it the people. Our ardent desire is to be the means of the greatest good to the largest number, rather than to live a life of self-interest and ease, and attract the largest share of laudatory encomiums.

We often do things that our best friends disapprove, even when going by our best light and judgment ; but then we must act by these, the suggestions and opinions of friends to the contrary notwithstanding ; and we sometimes receive a warm commendation for the performance of a plain, simple duty.

The REGISTER has no special pleading or bidding for friends, seeks to make no antagonists, and has no time or disposition to fight those who choose to place themselves in hostile atitude. Now we trust the future course of the REGISTER may be inferred from the above.

### TRANSACTIONS OF THE IOWA STATE DENTAL SOCIETY.

The eleventh annual meeting of this Society was held at Davenport, May 13—16, 1873.

We have received the Volume of Transactions, which contains much that is of great interest to the profession. The papers and discussions are of unusual interest.

We think it very desirable that every state society should publish its transactions in some permanent form. This is important, that the members may know, and that others may know, what progress is being made. It also serves as a stimulus to the members to press on to higher attainments; it also serve as a stimulus to other societies to do likewise.

Every progressive member of the profession should have copies of all these works.

This volume may be obtained by addressing Dr. A. V. Eaton, Anamosa, Iowa, who is the Secretary of the Society.

The next meeting will be held in Dubuque on the third Tuesday of May, 1874.

### TRANSACTIONS OF THE CALIFORNIA STATE SOCIETY.

We have before us this Volume of Proceedings, and a very interesting and instructive one it is, too. We cannot here enter into a special resume' of it, but will say that it is a volume that ought to be in the hands of every dentist in the country. The volumes of transactions ought to be and usually are the culmination and essence of dental science and practice, valuable to both practititioner and student. The views of the best men in the profession on all practical points are given in these volumes. This volume contains the papers, essays and proceedings of the annual meetings of the California State Dental Society for the years 1870—71—72 and 1873. All of which make a very valuable volume indeed, and we would advise every one to procure a copy and give it a thorough perusal.

Address Dr. N. J. Plomteaux, Woodland, Cal.

### TRANSACTIONS OF THE ILLINOIS STATE DENTAL SOCIETY.

The ninth annual meeting of the Illinois State Dental Society was held at Rock Island, May 13, 1873. Its proceedings, essays and discussions were promptly published, and have been at hand some time. This was the first state society that published its proceedings in a volume of transactions. This it has done for three years past.

The promptness and perfection with which the work has been done exhibits an energy and taste worthy of imitation. Some of the strongest men in the profession, in this country at least, are in Illinois and are members of this body. The correctness of this statement is quite apparent upon the perusal of this volume.

Its pages are rich—full of good things; and we suggest to every dentist who feels his need of more knowledge to add it to his library, which he can do by addressing Dr. E. R. E. Koch, of Chicago, Secretary of the Society.

### THE TABLE OF SOCIETIES.

In the presentation of the list of the dental societies in the United States, in each No. of the REGISTER, we desire it as full and complete as possible, and this it is impossible for us to do without the co-operation of the officers of the respective societies; either the recording or corresponding secretary. Now the list is really a standing card for all societies mentioned in it, and it is desirable on the part of all, and especially for particular societies, that they be correctly presented.

We propose in the next number to give, in addition to what is now presented, the number of active members belonging to each society, if the officers will promptly furnish them to us. We shall be glad to have a complete list of the names of the active members of every society in the country; this we desire as an aid in making up a report on dental associations for the next American Dental Association. We hope they may be furnished promptly by every society.

